# Progression in the In Vitro Macrophage Expansion

**DOI:** 10.1155/jimr/9994439

**Published:** 2025-04-28

**Authors:** Yunpeng Wei, Jingzhao Yang, Wenhong Zu, Mengran Wang, Yong Zhao

**Affiliations:** ^1^Faculty of Synthetic Biology, Shenzhen University of Advanced Technology, Shenzhen 518107, China; ^2^CAS Key Laboratory of Quantitative Engineering Biology, Shenzhen Institute of Synthetic Biology, Shenzhen Institute of Advanced Technology, Chinese Academy of Sciences, Shenzhen 518055, China

**Keywords:** in vitro, macrophages, proliferation, self-renewal

## Abstract

Macrophages play essential roles in homeostasis and disease, and they were considered terminally differentiated cells that cannot proliferate. However, growing evidence shows that macrophages can self-renew in homeostasis and multiple pathological states in vivo and artificial induction in vitro. With the rise of immune cell therapy based on macrophages, large-scale in vitro expansion of macrophages has become more and more urgent. However, the proliferation of macrophages in vitro is still inefficient because of the heterogeneity of macrophages, complicated crosstalk between macrophages and their microenvironments, and poor understanding of macrophage proliferation regulations. In this review, we summarized the discoveries known to stimulate macrophage proliferation in vitro, including cytokines, small molecule compounds, metabolites, the composition of pathogens and apoptotic cells, natural product extracts, gene editing, and other factors, as well as related mechanisms. It can be concluded that the promotion of macrophage proliferation in vitro covers various approaches and mechanisms. However, it is still necessary to test more strategies and learn more macrophage proliferation mechanisms to achieve large-scale engineering expansion of macrophages in vitro.

## 1. Introduction

Macrophages can be derived from embryonic precursor cells or bone marrow-derived circulating monocytes, which play pivotal roles in development, tissue homeostasis, and the response and resolution of inflammation after injury or infections. Macrophages were usually considered to be terminally differentiated cells that cannot self-renew, and they were replenished by bone marrow-derived circulating monocytes. However, more and more evidence demonstrated the proliferative potential of tissue-resident and monocyte-derived macrophages [1]. Macrophage proliferation has been observed in diverse organs and tissues such as the skin, peritoneum, lung, heart, aorta, kidney, liver, pancreas, brain, spinal cord, eye, adipose tissue, and uterus across different species like mice, rats, rabbit, and humans under homeostasis or pathological conditions [2]. For example, the supplementation of microglia and Langerhans cells is almost independent of blood monocytes derived from hematopoietic stem cells in the bone marrow [3]. In the case of atherosclerosis, local macrophages in arteriosclerotic vessels can proliferate about four times [4]. Moreover, other studies have shown that monocyte-derived macrophages can regain their proliferative capacity after entering tissues, thus being able to replenish the macrophage pool in tissues [5–7].

Meanwhile, with the progress of immune cell therapy based on macrophages such as chimeric antigen receptor macrophage (CAR-M) immunotherapy, macrophage reprogramming therapy, and several other therapies, large-scale in vitro expansion of macrophages has become increasingly urgent. However, the proliferation of mature macrophages in vitro is still inefficient because of the high heterogeneity of macrophages themselves and the poor understanding of the mechanisms and conditions required for their proliferation [8]. For this problem, immortalized cell lines, including THP1 and RAW 264.7 macrophages, are optional [9], but there is a risk of tumorigenesis. Another option, induced pluripotent stem cell-derived macrophages (iMACs), is to expand edited stem cells on a large scale and differentiate them into macrophages [10]. Nevertheless, this approach may have the problem of drift in cell characteristics after inducing differentiation. Therefore, direct and mass expansion of mature macrophages has unparalleled advantages. Thus, this review summarizes the studies known to stimulate mature macrophage proliferation in vitro and the corresponding mechanisms to provide a global perspective.

## 2. Cytokines

Cytokines play crucial roles in the proliferation, differentiation, migration, communication, and function execution of macrophages. Cytokines known to promote the proliferation of macrophages include macrophage colony-stimulating factor (M-CSF), granulocyte-macrophage CSF (GM-CSF), transforming growth factor *β* (TGF-*β*), insulin-like growth factor 1 (IGF-1), arachidonic acid and eicosapentaenoic acid-derived cytokines, and several combinations of cytokines. Their proliferative effects on macrophages and related mechanisms are summarized below, and more details are shown in Table 1.

### 2.1. M-CSF/GM-CSF

M-CSF and GM-CSF are classical cytokines that promote macrophage proliferation; related research has been ongoing since the 1980s and continues to this day. Chen et al. [11] reported that GM-CSF can promote the proliferation and clonal growth of murine peritoneal exudate macrophages (PEMs) and bone marrow-derived macrophages (BMDMs). Meanwhile, GM-CSF greatly enhances the responsiveness of the two kinds of cells to M-CSF. Moreover, it was discovered that M-CSF (1%–50% L929-conditioned medium [CM]) and 1%–50 U/mL recombinant murine GM-CSF (rMuGM-CSF) can promote the proliferation of liver macrophages in rats in a concentration-dependent manner [12]. Fejer et al. [13] reported that they obtained GM-CSF/signal transducer and activator of transcription 5-dependent macrophages named Max Planck Institute cells (MPI) by culturing unseparated fetal liver cells for about 8 weeks in the presence of GM-CSF (usually 30 ng/mL), which are predominantly round adherent cells similar to alveolar macrophages (AMs). These cells can exponentially proliferate to 10^4^ folds when cultured in vitro in the presence of 30 ng/mL GM-CSF for about 17 days. Ito et al. [14] discovered that one marrow-derived precursor cell could become a group of macrophages with proliferation potential and maintain the phenotype and function of macrophages without tumorigenicity under a long-term culture of GM-CSF (3 months), named GM-CSF-dependent self-renewable immature macrophages (GM-IMs). The cell number of GM-IMs increased to approximately 6 folds compared to the control after 6 days of culture in a CM containing 10% GM-CSF. Ivanov et al. [15] found that M-CSF secreted by mesothelial cells promotes the proliferation of peritoneal macrophages and BMDMs in vitro, which is entirely CSF1R dependent.

A mechanistic study revealed that M-CSF-mediated ROS generation leads to the oxidation of SHP1, thereby promoting macrophage proliferation through a PI3K/Akt-dependent signaling pathway [16]. In addition, a recent study found that GM-CSF contributes to the proliferation and self-renewal of macrophages by ensuring proper mitochondrial functions, including promoting fatty acid beta-oxidation and significantly enhancing tricarboxylic acid cycle activity, oxidative phosphorylation, and ATP production [17]. Taken together, M-CSF and GM-CSF may still be the basic cytokines in the in vitro macrophage expansion, which could effectively expand macrophages if combined with other cytokines or other components.

### 2.2. IGF-1

IGF-1 is a multifunctional growth factor with a similar structure to insulin, mediating growth promotion and metabolic regulation [26]. O'Donnell et al. [18] found that 50 ng/mL IGF-1 can significantly promote DNA synthesis in adult rat brain microglia, the macrophages in the central nervous system, in vitro. However, related mechanisms are still unknown. Thus, IGF-1 may be useful when expanding microglia in vitro.

### 2.3. Arachidonic Acid and Eicosapentaenoic Acid-Derived Cytokines

The metabolites of arachidonic acid and eicosapentaenoic acid are various, and some of them are identified as functional cytokines that can promote the proliferation of macrophages. Nieves and Moreno investigated the effects of prostaglandin E2 (derived from arachidonic acid)/prostaglandin E3 (derived from eicosapentaenoic acid) (PGE_2_/PGE_3_), and leukotriene B4 (derived from arachidonic acid)/leukotriene B5 (derived from eicosapentaenoic acid) (LTB_4_/LTB_5_) on the proliferation of RAW 264.7 macrophages. It was discovered that PGE_2_/PGE_3_ (1–10 nM), LTB_4_ (10–100 nM)_,_ and LTB_5_ (100 nM) can promote the proliferation of RAW 264.7 macrophages in the absence of other growth factors [19]. The same research group further observed that LTB_4_ and LTD_4_ promote the proliferation of RAW 264.7 macrophages in the absence of growth factors and demonstrated that this effect may be achieved through the mitogen-activated protein kinase (MAPK) and phosphatidylinositol 3-kinase (PI3K) pathways [27].

### 2.4. Combined Cytokines

The proliferative impact of a single cytokine on macrophages is often limited, whereas the combined administration of multiple cytokines could exhibit remarkable synergistic effects.

*M-CSF or GM-CSF with TGF-β* Fan et al. [20] reported that 0.1–1.0 ng/mL recombinant human TGF-*β*1 (rHuTGF-*β*1) significantly enhanced PEM growth in response to rMuGM-CSF stimulation. Similar effects were observed with rHuTGF-*β*1 in murine AMs and BMDMs. rHuTGF-*β*1 treatment significantly enhanced GM-CSF receptor expression in PEM, which was time- and dose-dependent, suggesting that rHuTGF-*β*1 may have a synergistic mechanism when combined with rMuGM-CSF. Dobbertin et al. [21] also discovered that TGF-*β*2 produced by neurons can promote the proliferation of rat microglia and BMDMs in the presence of M-CSF (20% L9CM). In addition, it was found that rHuTGF-*β*1 or *β*3 (1 × 10^−4^ ~ 1 ng/mL) alone does not support the survival of rat macroglia or BMDMs but markedly enhances 10 ng/mL M-CSF-induced proliferation of these cells. Luo et al. [22] recently reported that mouse bone marrow and fetal liver cells could be induced into alveolar macrophage-like cells by supplementing murine GM-CSF, human TGF-*β*, and PPAR*γ* agonists rosiglitazone (GTR). Moreover, the GTR medium can promote the proliferation of AMs isolated from bronchoalveolar lavage fluid (BALF-AMs).

*M-CSF and TNF-α* Branch et al. [23] reported that *TNF-α* (≥1250 ng/mL) alone only slightly stimulates the proliferation of macrophages. However, the combination of TNF-*α* (125 ng/mL) and M-CSF (25 or 100 U/mL) stimulates the proliferation of BMDMs and the clonal growth factor-dependent macrophage line (S1) derived from C3H/HeJ bone marrow cells. *TNF-α* was found to temporarily down-regulate the levels of M-CSF receptors on both cell lines. However, after 24 h of incubation, the receptor numbers were restored to the initial level or higher. Further study revealed that the synergistic proliferative effects of TNF-*α* on macrophages are mediated by the TNF receptor of 55–60 kD, which can directly act on proliferative macrophages to significantly reduce the cell doubling time and enhance the promacrophage proliferative effect of M-CSF [24].

*IL-33 and M-CSF* IL-33 can promote the proliferation of BMDMs induced by M-CSF in vitro, which depends on the ST2 receptor, but the specific pathway is unclear [25].

## 3. Small Molecule Compounds

Small molecule compounds with specific pharmacological or toxicological properties are usually potential drug candidates, which can be artificially synthesized or extracted and purified natural products. Small molecule compounds that have been discovered so far to promote macrophage proliferation mainly include erythromycin, dexamethasone, bisphenol A (BPA), and metformin. More details are shown in Table 2.

### 3.1. Erythromycin

As a classic antibiotic, the proliferative effect of erythromycin on macrophages is little known. Kita et al. [28] discovered that erythromycin (0.2–20 mg/mL) can remarkably induce the proliferation of murine PEMs without exogenous growth factors, resulting in huge colonies between days 22 and 26 of culture. Surprisingly, these colonies continue to proliferate even after subculture. Further investigation demonstrated that this effect may be related to the direct action of erythromycin itself. The molecular mechanisms involved have not been uncovered, which may involve the immortalized transformation of macrophages.

### 3.2. Dexamethasone

Lloberas et al. [29] reported that dexamethasone (10^−7^ ~ 10^−6^ M), one of the classic steroids, does not affect the proliferation of bone marrow-derived mature or immature macrophages. However, dexamethasone enhances the proliferation of these cells in the presence of M-CSF or GM-CSF in vitro. This effect may be related to the up-regulation of glucocorticoid receptors and increased autocrine production of M-CSF.

### 3.3. BPA

Ampem et al. [30] discovered that BPA (1 and 10 nM), an organic environmental pollutant and endocrine disruptor, can enhance the self-renewal of J774A.1 macrophage and adipose tissue macrophages (ATMs) by the activation of ERK/MAPK signaling and a slight elevation in liver X receptor *α* (LXR*α*) expression. However, considering the toxicity of BPA, it is unlikely to be a candidate agent for in vitro macrophage expansion.

### 3.4. Metformin

Feng et al. [31] found that 0.5 mM metformin, an antidiabetic drug with multiple pharmacological functions, facilitated proliferation and inhibited apoptosis of macrophages pretreated with 50 μg/mL oxidized low-density lipoprotein (Ox-LDL), which may be related to the inhibition of miRNA-34a and elevated expression of Bcl2 [32].

## 4. Metabolites

Metabolites generated during physiological or pathological processes are critical indicators to assess health status or disease progression. Meanwhile, some of these metabolites may also alter the proliferation characteristics of macrophages. It has been discovered that Ox-LDL, urea, and advanced glycation end products (AGEs) can simulate the proliferation of macrophages in vitro, which could be helpful in expanding macrophages in vitro. More details can be seen in Table 3.

### 4.1. Ox-LDL

Elevated Ox-LDL levels in plasma are usually a critical marker in the initiation and development of atherosclerosis [44]; however, according to existing research, Ox-LDL can also induce macrophage proliferation in vitro. Sakai et al. [33, 34] reported that the growth of murine and human macrophages could be induced by Ox-LDL, in which the main phospholipid component, lysophosphatidylcholine (lyso-PC), plays a key role. Further study demonstrated that macrophage scavenger receptor (MSR) is an essential and efficient pathway for internalizing lipid PC degradation products in Ox-LDL-induced macrophage proliferation [35]. Moreover, Matsumura et al. [36] uncovered that Ox-LDL rapidly induces a transient increase in intracellular free calcium ions and activates membrane protein kinase C (PKC). Thus, there are two intracellular signaling pathways that activate PKC: one is mediated by G proteins sensitive to pertussis toxin-induced calcium elevation, and another is mediated by MSR-mediated uptake of lyso-PC. Biwa et al. [37] revealed that effective endocytosis of lyso-PC of Ox-LDL by macrophages through MSR-AI/AII and subsequent PKC activation led to increased autocrine GM-CSF release into the medium, resulting in enhanced macrophage proliferation; and PI3K is involved in at least part of the downstream signaling pathway, acting after GM-CSF induction [38]. Besides, the expression of group-II PLA_2_ may also play an essential role in Ox-LDL-induced macrophage proliferation by stimulating the GM-CSF release [39]. In addition, Brunner et al. [40] reported that Ox-LDL can promote LPS-interferon-stimulated mouse J774.A1 macrophage proliferation by inhibiting the expression of inducible nitric oxide synthase (iNOS) at both mRNA and protein levels, resulting in decreased nitric oxide (NO) production and subsequently reduced NO-induced apoptosis in macrophages.

### 4.2. Urea

Moeslinger et al. [41] and Moeslinger and Spieckermann [42] found that 60–150 mM urea, the end product of protein metabolism, promotes the proliferation of 1 μg/mL LPS-activated RAW 264.7 macrophages. Urea suppressed iNOS-dependent NO production, resulting in reduced apoptosis mediated by NO.

### 4.3. AGEs

AGEs are formed nonenzymatically by covalent binding between carbonyl groups of reducing sugars and free amine groups of nucleic acids, proteins, or lipids. Further rearrangements eventually generate stable and irreversible end-products, which are considered to take part in many pathophysiological processes and diseases like diabetes [45]. Recently, a study investigated the proliferative effects of glycolaldehyde-derived AGEs (Glycol-AGEs) on the RAW 264.7 macrophages. The results showed that Glycol-AGEs (1–10 μg/mL) significantly promoted the proliferation of RAW 264.7 macrophages in a dose-dependent manner via the JAK-STAT pathway [43].

## 5. Composition of Pathogens and Apoptotic Cells

Recognition and elimination of pathogens and apoptotic cells are the essential functions of macrophages, and they may trigger the proliferation of macrophages under some conditions. *Mycoplasma* membranes, type B CpG oligodeoxynucleotides (CpG-B ODNs), and apoptotic Jurkat and NIH 3T3 cells were discovered to stimulate macrophage proliferation, which could be a potential approach to expand macrophages in vitro. More details can be seen in Table 4.

### 5.1. Composition of Pathogens

Stuart et al. [46] investigated the proliferative effects of membranes derived from three *mycoplasma* species, including *M. arginine*, *M. arthritidis*, and *M. pulmonis*, on BMDMs. The results showed that the membrane from *M. arginine* and *M. arthritidis* could significantly promote the proliferation of BMDMs instead of that from *M. pulmonis*, and the proliferative effect seems dependent on GM-CSF production. Chen et al. [47] discovered that CpG-B ODNs, unmethylated CpG dinucleotides present in bacterial DNA that can be recognized by pattern recognition receptor Toll-like receptor 9 (TLR9), such as CpG-ODN 1668, can promote macrophage proliferation and migration by inducing interleukin 1 receptor antagonist (IL-1Ra) and down-regulating the expression of p27. Further investigation revealed that the induction of IL-1Ra by CpG-B ODNs was F-spondin dependent.

### 5.2. Apoptotic Cells

Gerlach et al. [48] discovered that efferocytosis of apoptotic Jurkat cells (ACs) can significantly induce the proliferation of murine BMDMs and human macrophages in vitro. Moreover, mechanism research showed that there are two pathways involved: (1) an ERK1/2 pathway activated when ACs engage the macrophage MerTK receptor; (2) nucleotides generated from the degradation of apoptotic cell DNA through phagolysosomal DNase2a activation initiate a signaling pathway involving DNA-PKcs-mTORC2/Rictor. These two pathways ultimately lead to upregulating Myc and Bhlhe40 expression and suppressing *c-Maf*, resulting in elevated proliferation of noninflammatory macrophages. Further investigation demonstrated the second mechanism in the apoptotic cells induced macrophage proliferation: lactic acid promotes the stability of Myc protein and subsequent macrophage proliferation through the G-protein-coupled receptor 132 (GPR132)-PKA-AMPK-NAD^+^-NADH-SIRT1-Myc signaling pathway. Taken together, efferocytosis-induced macrophage proliferation requires two independent processes: a signaling pathway induced by apoptotic cell-derived nucleotides and a cellular metabolism pathway involving lactate production [49, 50]. In addition, Knuth et al. [51] showed that apoptotic cells can induce remarkable M2-like peritoneal macrophage proliferation. However, such higher levels of proliferation are not related to either through phagocytosis of apoptotic bodies or the released mediators. GSEA and GO analysis showed enrichment of the PEG_2_, cell cycle, and DNA replication pathways.

## 6. Natural Product Extracts

Like small molecular compounds, natural product extracts could also be candidates for drugs or biomedical engineering materials. Among them, it was discovered that heparin and heparan sulfate, hyaluronan, ceramide 1-phosphate, and fungal polysaccharides derived from *Lactarius deliciosus* (L. ex Fr.) Gray, etc., can promote the proliferation of macrophages, which could be a potential strategy to expand macrophages in vitro. More details are shown in Table 5.

### 6.1. Heparin and Heparan Sulfate

Heparin and heparan sulfate are common natural anticoagulants. Sorimachi et al. [52] discovered that heparin (5–50 μg/mL) and heparan sulfate (10 μg/mL) could induce macrophage proliferation. Blockage of fibroblast growth factor or GM-CSF with their antibodies did not prevent macrophage proliferation with heparin, suggesting other unknown mechanisms. Meanwhile, it was found that polyanions such as lignin derivatives and dextran could also remarkably promote the multinucleation and proliferation of rat BMDMs at 10 μg/mL.

### 6.2. Hyalgan

Hyalgan, or hyaluronic acid, is a disaccharide unit glycosaminoglycan composed of D-glucuronic acid and N-acetylglucosamine, which is a biochemical drug with high clinical value that is widely used in various eye surgeries, such as lens implantation, corneal transplantation, and antiglaucoma surgery. It can also be used to treat arthritis and accelerate wound healing [59]. Sheehan et al. [53] discovered that 0.01–0.1 mg/mL Hyalgan (500–730 kDa) significantly promoted U937 human macrophage proliferation and increased the proportion of G2/M cells without affecting the number of apoptotic or dead cells. However, 1 mg/mL Hyalgan significantly inhibited cell proliferation and altered the cell cycle distribution, leading to a decrease in the proportion of G0/G1 cells and an increase in the proportion of S and G2/M cells. These results show that the concentration of Hyalgan has different effects on the growth dynamics of macrophages.

### 6.3. Ceramide 1-Phosphate

Ceramide-1-phosphate (C1P) is a bioactive phosphorylated sphingolipid synthesized by ceramide kinase, which plays important roles in regulating cell survival, migration, apoptosis, and autophagy [60]. Gangoiti et al. [54] discovered that 50 μM C1P can promote the proliferation of murine BMDMs by activating the PI3-K/PKB, ERK, and JNK pathways to stimulate DNA synthesis. Furthermore, downstream effectors such as GSK-3*β*, *c-Myc*, cyclin D1, and NF-*κ*B were identified as significant contributors to this effect.

### 6.4. Fungal Polysaccharides

Fungi have been widely used in China as a part of the daily diet and traditional Chinese medicine, and some bioactive polysaccharides extracted from them are further identified as candidates for modern clinical drugs [61]. It was reported that polysaccharides (2.5–15 μg/mL) derived from *L. deliciosus* (L. ex Fr.) Gray, one of the edible fungi, can enhance the proliferation of RAW 264.7 macrophages by facilitating cell cycle progression in the G0/G1 phase and preventing cell cycle arrest in the G2/M phase. KEGG pathway and GO enrichment analysis revealed significantly differentially expressed gene enrichment in signaling pathways, including JAK/STAT, MAPK, chemokine, vascular endothelial growth factor (VEGF), and TGF-*β* [55].

### 6.5. Others

Hui et al. [56] reported that the exogenous addition of adiponectin, one of the most widely studied adipokines [62], can promote the proliferation of IL-4-polarized M2 macrophages by activating the intracellular PI3K-Akt pathway by binding to T-cadherin. Osteopontin, a highly phosphorylated glycol-phosphoprotein with multiple biological functions [63], can promote the proliferation of human monocyte-derived macrophages (human monocytes were isolated from peripheral blood by using density gradient centrifugation and separated with a CD14-positive magnetic activated cell sorting-MACS, which were further differentiated to M2-like macrophages for 6 days with 50 ng/mL M-CSF in normal RPMI 1640 medium) and mouse BMDMs in vitro. Related mechanisms were undetected [58]. Meanwhile, neuropeptide FF (NPFF), known as a morphine-modulating peptide capable of mediating the pain response [64], can promote the proliferation of ATMs by inhibiting the transcription of MafB [57].

## 7. Gene Editing

Except for the studies above, editing genes controlling self-renewal and cell cycle were also proven to stimulate macrophage proliferation effectively (Table 6). Baumbach et al. [65] discovered that bone marrow cells infected by *c-Myc* carrying murine retrovirus (MRV) partially transformed into mononuclear phagocytic-like cells, which gained extensive long-term proliferation capability in the presence of M-CSF in vitro. However, these cells are probably tumorigenic in vivo because *c-Myc* carrying MRV induced monocytic tumors in BALB/c mice after injection, and a high density of transformed cells with monocytic morphology and expression characteristics were found in their ascites fluid. Aziz et al. [66] reported that MafB/c-Maf-deficiency (Maf-DKO) allowed mature macrophages to proliferate extensively in vitro without losing their differentiated phenotype and function. Meanwhile, these expanded cells did not display oncogenicity and helped to form functional macrophage populations in vivo after transplantation. Mechanistic studies showed that the sustained proliferation of Maf-DKO macrophages depended on the upregulation of KLF4 and *c-Myc*. However, the potential tumorigenicity of gene-edited macrophages should be excluded before conducting any clinical trials.

## 8. Other Factors

### 8.1. Hyperoxia

Nerurkar et al. [67] revealed that AMs cultured in a high oxygen environment (95% oxygen +5% carbon dioxide) for 18 h showed increased uptake of ^3^H thymidine and proliferation in vitro compared to those cultured in a control environment (95% air +5% carbon dioxide). The specific mechanisms are unclear. Thus, a hyperoxia culture environment may be helpful to expand AMs in vitro.

### 8.2. Cadmium Ions

Misra et al. [68] reported that cadmium ions (Cd^2+^) significantly increased cell division of murine macrophages at micromolar concentrations (0.1–1 μM). Specifically, after overnight incubation, 0.1, 0.2, 0.5, and 1 μM Cd^2+^ increased the cell number to ~1.52, 1.73, 1.64, and 1.53 folds, respectively. Surprisingly, even after culturing for more than 4 weeks, macrophages treated with cadmium continued to proliferate. Further study indicated that the effects of Cd^2+^ were mediated by the p21^ras^-dependent MAPK pathway, which increases the availability of transcription factor NF-*κ*B and activates early genes *c-fos* and *c-myc*. However, considering that the proliferative effect of Cd^2+^ on macrophages could be a sign of induced immortalization and that Cd^2+^ itself is a toxic heavy metal ion, the tumorigenicity and Cd^2+^ contamination of expanded macrophages should be excluded if they were used for further studies.

## 9. Recent Discoveries on Intracellular Signals Regulating Macrophage Proliferation

**Figure 1:**
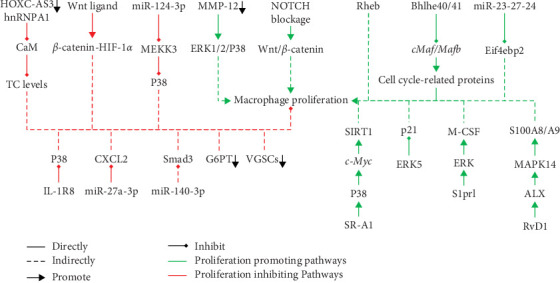
Summary of recent discoveries on macrophage proliferation regulation signaling pathways.

Understanding more intracellular signaling regulations of macrophage proliferation is essential for developing more effective strategies to expand macrophages in vitro. Here, we summarized recent discoveries on intracellular signals regulating macrophage proliferation to provide potential insights (Figure 1).

### 9.1. Signal Pathway Proteins

Guan et al. [69] found that MMP-12 knockdown promotes the proliferation of RAW 264.7 macrophages by activating the ERK/P38 MAPK signaling pathway. NOTCH blockade, however, suppressed the differentiation of monocyte-derived tumor-associated macrophages (TAMs), but promoted the proliferation of Kupffer cell-like TAMs in hepatocellular carcinoma and hepatic metastasis of colorectal cancer by upregulating Wnt/*β*-catenin signaling [70]. Zhang et al. [71] discovered that macrophage Ras homolog enriched in brain 1 (Rheb) plays an important role in Western diet-induced atherosclerosis by promoting macrophage proliferation, inflammation, and lipid uptake. However, the specific mechanisms are unclear. Giurisato et al. [72] found that ERK5 maintained the capacity of macrophages to proliferate by suppressing p21 expression to inhibit their differentiation. Zhu et al. [73] demonstrated that Wnt treatment stimulates AM proliferation by promoting *β*-catenin-HIF-1 *α* interaction and glycolysis-dependent inflammation while suppressing mitochondrial metabolism [74]. Ye et al. [75] reported that resolvin D1 (RvD1) improves resident AM self-renewal via the ALX/MAPK14/S100A8/A9 pathway in acute respiratory distress syndrome.

### 9.2. Receptors and Transport Proteins

Sun et al. [76] reported that the blockade of voltage-gated sodium channels (VGSCs) suppressed the proliferation in the RAW 264.7 macrophages, but the specific mechanisms are unclear. Zhang et al. [77] found that inhibition of class A1 scavenger receptor (SR-A1) suppressed the proliferation of cardiac resident reparative macrophages via the SR-A1-P38-SIRT1 (Sirtuin 1) pathway mediated by *c-Myc*. Moreover, Guo et al. [78] discovered that interleukin-1 receptor 8 (IL-1R8) suppresses macrophage proliferation by inhibiting the p38 MAPK signaling pathway. Jeon et al. [79] revealed that glucose-6-phosphate transporter (G6PT)-deficient macrophages exhibited a significant decline in cell growth, bactericidal activity, and antiviral response, suggesting that G6PT-mediated metabolism is essential for effector functions of macrophages. In addition, Kuang et al. [80] demonstrated that the endothelial sphingosine 1-phosphate receptor 1 (S1pr1) promoted Ly6c^low^ macrophage proliferation in a cell-contact manner by activating the ERK signaling pathway and enhancing CSF1 expression.

### 9.3. Transcription Factor

Jarjour et al. [81] reported that the transcription factor Bhlhe40 repressed the expression of *c-Maf* and *Mafb* and directly promoted the expression of cell cycle-related proteins to promote the proliferation of large peritoneal macrophages, but not that of other tissue-resident macrophages. Rauschmeier et al. [82] identified the transcription factors Bhlhe40 and Bhlhe41 that can promote the proliferation of AMs by directly repressing the expression of lineage-inappropriate genes in AMs.

### 9.4. MicroRNA and Long Noncoding RNA

Zhai et al. [83] reported that miR-124-3p overexpression downregulated MEKK3 expression and inhibited the expression of the p38MAPK signaling pathway, thereby inhibiting macrophage proliferation and promoting macrophage apoptosis in mice with coronary AS. Qiao et al. [84] demonstrated that elevated miR-140-3p decreased the proliferation rates of RAW 264.7 macrophages, while suppressed miR-140-3p increased the proliferation rates of RAW 264.7 macrophages. Further study suggested that Smad3 might be the target gene of miR-140-3p. Sheng et al. [85] discovered that the knockdown of HOXC-AS3, a long-chain noncoding RNA, led to a decrease in the proliferation, colony formation, invasiveness, and tumorigenicity of tumor macrophages via the HOXC-AS3/hnRNPA1/CaM pathway in glioblastoma. Besides, Sprenkle et al. [86] revealed that miR-23-27-24 clusters can promote the proliferation of macrophages by directly targeting the mRNA of *Eif4ebp2*, a gene that restricts protein synthesis and proliferation in macrophages. In addition, Zhai et al. [87] demonstrated that miRNA-27a-3p suppresses the proliferation of the pulmonary macrophage by downregulating the expression of CXCL2 in nonsmall cell lung cancer.

## 10. Summary and Perspective

Macrophages play crucial roles in maintaining immune homeostasis in vivo with unique properties like massive infiltration and enhanced phagocytosis; it is foreseeable that they have great potential for application in immune cell therapy like CAT-M and many other therapies for the treatment of solid tumors. However, the limited proliferation of macrophages in vitro hindered the large-scale expansion of engineered macrophages and their therapeutic clinical applications [88]. No doubt, terminally differentiated macrophages can still self-renew and proliferate under certain homeostatic or pathological conditions, but constructing proper culture environments in vitro to activate their proliferation potential is an urgent problem that needs to be solved. This review summarizes the discoveries known to stimulate macrophage proliferation in vitro, including cytokines, small molecule compounds, metabolites, the composition of pathogens and apoptotic cells, natural product extracts, gene editing, and other factors, as well as related mechanisms. However, due to the heterogeneity of macrophages and complicated crosstalk between macrophages and their microenvironments, these discoveries are far from being able to expand mature macrophages in vitro massively, but they are valuable footstones. Based on these discoveries, it is possible to find more effective strategies, which could be entirely new substance components, gene editing strategies, or ingenious combinations of existing approaches. Besides, recent studies have revealed some new mechanisms of macrophage proliferation regulation, and it may be possible to stimulate macrophage proliferation in vitro via targeting relevant signaling pathways by gene editing or metabolic reprogramming to achieve industrial-scale expansion of macrophages. However, to meet the urgent demand for industrial-scale expansion of macrophages, more fundamental research on the regulation of macrophage proliferation is still indispensable. With the establishment of more efficient protocols for expanding mature macrophages in vitro, macrophage-based clinical therapy will enter a new Era.

## Figures and Tables

**Table 1:** Proliferative effects of cytokines on macrophages and related mechanisms.

Cytokines	Treatment protocols	Results	Mechanisms	References
GM-CSF	1500 U/mL GM-CSF were used to treat murine PEMs and BMDMs for 0–9 days, respectively. Meanwhile, the two types of cells were treated with 1200 U/mL M-CSF and 30 U/mL GM-CSF for 0–10 days	1. 1500 U/mL GM-CSF increased the cell number of PEMs and BMDMs to ~14.23 (on day 9) and ~21.46 (on day 5) folds, respectively; 2. 30 U/mL GM-CSF + 1200 U/mL M-CSF increase the cell number of PEMs and BMDMs to ~4.07 and ~1.99 folds compared to 1200 U/mL M-CSF treatment alone, respectively	1. GM-CSF may directly stimulate macrophage proliferation; 2. GM-CSF enhances the macrophage responsiveness to M-CSF	[11]

M-CSF + GM-CSF	Rat liver macrophages were treated with M-CSF (0%–50% L929 conditioned medium) and rmGM-CSF (0–100 U/mL) for 7 days, respectively	M-CSF (50% L929-CM) and 50 U/mL GM-CSF promoted the incorporation of [^3^H] dThd by rat liver macrophages to ~12.5 and ~4.98 folds, respectively	Not mentioned	[12]

GM-CSF	AMs like MPI cells were prepared and treated with 30 ng/mL murine GM-CSF for about 17 days	MPI cells grow exponentially to ~100 and ~100,000 folds compared to the M-CSF treatment and the control group, respectively	GM-CSF-induced STAT5 confers self-renewing capacity to MPI cells and maintains their innate reactivity	[13]

GM-CSF	GM-IMs were continuously cultured with 10% GM-CSF conditioned medium (CM) for 6 days	In 10% GM-CSF-CM, the cell number of GM-IMs increased to ~6 times compared to the control	Not mentioned	[14]

M-CSF	100,000 peritoneal macrophages were co-cultured with 200,000 M-CSF-producing mesothelial cells for 8 days	The number of macrophages increased to ~26.42 folds after co-culturing with mesothelial cells	M-CSF produced by mesothelial cells induced the proliferation of peritoneal macrophages	[15]

M-CSF	Murine BMDMs were treated with 0–30 ng/mL of M-CSF for 48 h	5, 15, and 30 ng/mL M-CSF promoted the BrdU incorporation of BMDMs to ~2.24, ~3.74, and ~3.81 folds, respectively	Via ROS-mediated oxidation of SHP1 and PI3K/Akt-dependent signaling pathways	[16]

GM-CSF	Murine BMDMs were treated with a CGM medium containing 10 ng/mL GM-CSF for 2 h	GM-CSF promoted the EdU incorporation of macrophages to ~3 folds	Ensuring proper mitochondrial functions	[17]

IGF-1	Adult rat brain microglia were treated with 5 ng/mL insulin and 50 ng/mL IGF-1 for 24 h	DNA synthesis in adult rat brain microglia increased to ~2 folds	Not mentioned.	[18]

PGE_2_/PGE_3_, LTB_4_/LTB_5_	Without other growth factors, RAW 264.7 macrophages were treated with PGE_2_/PGE_3_ (1, 10 nM), and LTB_4_/LTB_5_ (10, 100 nM) for 48 h, respectively	1 and 10 nM PGE_2_, 1 and 10 nM PGE_3_, 10 and 100 nM LTB_4_, and 100 nM LTB_5_ promoted the proliferation of RAW 264.7 macrophages to ~1.83, ~1.6, 2.01, ~1.83, ~1.34, ~1.71, and ~1.41 folds, respectively	Not mentioned	[19]

LTB_4_/Ltd_4_	Without other growth factors, RAW 264.7 macrophages were treated with 0.001–0.01 μM LTB_4_ and Ltd_4_ for 48 h, respectively	0.01 μM LTB_4_ and 0.01 μM Ltd_4_ promote the proliferation of RAW 264.7 macrophages to ~2.31 and ~1.6 folds, respectively	Probably through the MAPK and PI3K pathways	[19]

rHuTGF-*β*1+rMuGM-CSF	PEMs, AMs, and BMDMs were treated with 0.1–1.0 ng/mL rHuTGF-*β*1 combined with 0.5 ng/mL rMuGM-CSF for 7–9 days	1. 1.0 ng/mL rHuTGF-*β* combined with 0.5 ng/mL rMuGM-CSF increased the proliferation of PEM to ~3 folds compared to 0.5 ng/mL rMuGM-CSF alone. 2. Similar effects were observed with 1.0 ng/mL rHuTGF-*β*1 combined with 0.5 ng/mL rMuGM-CSF on murine AMs and BMDMs (~1.64 and ~1.46 folds, respectively)	Significantly enhanced GM-CSF receptor expression	[20]

rhTGF-*β*1/*β*2/*β*3+ rhM-CSF	Rat microglia and BMDMs were treated with rHuTGF-*β*1/*β*2/*β*3 (0–1 ng/mL) in the presence of 10 ng/mL rHuM-CSF, for 96 h, respectively	1. rHuTGF-*β*1/*β*2/*β*3 promoted the proliferation of rat microglia up to ~1.48 (0.1 ng/mL rHuTGF-*β*1), ~1.53 (1 ng/mL rHuTGF-*β*2), and ~1.47 (0.1 ng/mL rHuTGF-*β*3) folds, respectively; 2. rHuTGF-*β*1/*β*2/*β*3 promoted the proliferation of BMDMs up to ~1.95 (0.1 ng/mL rHuTGF-*β*1), ~1.74 (0.1 ng/mL rHuTGF-*β*2), and ~2.01 (1 ng/mL rHuTGF-*β*3) folds, respectively	Not mentioned	[21]

GM-CSF, TGF-*β*, and PPAR*γ* agonists	Murine BALF-AMs were cultured in a medium containing 20 ng/mL GM-CSF, 2 ng/mL TGF-*β*, and 0.1 μM PPAR-*γ* agonist rosiglitazone (GTR) for 9 days	This GTR medium promoted 2.5 × 10^4^, 5 × 10^4^, and 10 × 10^4^ BALF-AMs to proliferate to ~ 28.69, ~20.875, and ~ 8.94 folds, respectively	By activating self-renewal genes such as *Klf4* and *Myc* via suppressing *Maf* and *Mafb*	[22]

M-CSF + TNF-*α*	BMDMs and the clonal growth factor-dependent macrophage line (S1) derived from C3H/HeJ bone marrow cells were treated with 125–1250 ng/mL TNF-*α* and 25–2500 U/mL M-CSF for 2 days	1. TNF-*α* (1250 ng/mL) + M-CSF (2500 U/mL) stimulated the proliferation of BMDMs and the S1 cell line to ~26.32 and ~411.5 folds, respectively; 2. 125 ng/mL TNF-*α* + 25 U/mL M-CSF increased the proliferation of BMDMs to ~19.7 folds; 125 ng/mL TNF-*α* + 100 U/mL M-CSF increased the proliferation of S1 cell line to ~183.3 folds	TNF-*α* temporarily down-regulates the levels of M-CSF receptors on both cell lines	[23]

M-CSF + TNF-*α*	Macrophages (S1 cell line) were treated with 100 ng/mL hTNF-*α* and 5000 U/mL M-CSF for 9 h	TNF-*α* and M-CSF stimulate the ^3^HTdR uptake of the S1 cell line to ~49.89 folds	Mediated by the TNF receptor of 55–60 kD, which can directly act on proliferative macrophages	[24]

IL-33 and M-CSF	BMDMs were treated with 25 ng/mL M-CSF and 0–10 ng/mL of IL-33 for 3 days	The percentage of S and G2 cells increased to ~1.37 folds by 2.5 ng/mL IL-33 combined with 25 ng/mL M-CSF	IL-33-ST2 axis contributes to the proliferation	[25]

**Table 2:** Proliferative effects of small molecule compounds on macrophages and related mechanisms.

Small molecule compounds	Treatment protocols	Results	Mechanisms	References
Erythromycin	Murine PEMs were treated with erythromycin (0.2–20 mg/mL) (full text is unavailable)	Erythromycin remarkably induced the proliferation of murine PEMs without exogenous growth factors (full text is unavailable)	Unclear	[28]

Dexamethasone	BMDMs were treated with dexamethasone (10^−7^ ~ 10^−6^ M) alone or dexamethasone (10^−7^ ~ 10^−6^ M) combined with M-CSF (1200 U/mL) or GM-CSF (0–20 mg/mL) for 7 days	1. Dexamethasone alone did not affect the proliferation of BMDMs; 2. 10^−6^ M dexamethasone enhanced the thymidine incorporation of BMDMs in the presence of M-CSF (1200 U/mL) or GM-CSF (5–20 mg/mL) in vitro to ~1.75 and ~1.20 folds compared to M-CSF or GM-CSF alone, respectively	This effect may be related to the up-regulation of glucocorticoid receptors and increased autocrine production of M-CSF	[29]

Bisphenol A	J774A.1 macrophages and ATMs were treated with BPA (1 and 10 nM) for 18 h	10 nM BPA enhanced the self-renewal of J774A.1 macrophage and ATMs to ~1.16 and ~1.38 folds, respectively	By activating ERK/MAPK signaling and slightly elevating liver X receptor *α* (LXR*α*) expression	[30]

Metformin	THP1 macrophages pretreated with 50 μg/mL Ox-LDL were treated with 0.5 mM metformin for 0–72 h	After 72 h, 0.5 mM metformin facilitated proliferation to ~1.58 folds and inhibited apoptosis by ~53% of macrophages pretreated with 50 μg/mL Ox-LDL	The inhibition of miRNA-34a and elevated expression of Bcl2	[31]

**Table 3:** Proliferative effects of Metabolites on macrophages and related mechanisms.

Metabolites	Treatment protocols	Results	Mechanisms	References
Ox-LDL	Murine peritoneal macrophages were treated with 0–50 μg/mL Ox-LDL for 0–10 days	After 6 days of treatment,10, 20, and 50 μg/mL Ox-LDL enhanced the proliferation of peritoneal macrophages to ~5.2, ~8.5, and ~11.6 folds, respectively	Lyso-PC plays an essential role	[33]

Ox-LDL	Human peripheral blood monocyte-derived macrophages were treated with 0–50 μg/mL Ox-LDL for 0–4 days	After 4 days of treatment,10, 20, and 50 μg/mL Ox-LDL enhanced the proliferation of macrophages to ~1.75, ~2.16, and ~2.39 folds, respectively	Lyso-PC plays an essential role	[34]

Ox-LDL	Murine peritoneal macrophages were treated with 10 μg/mL Ox-LDL for 6 days	Ox-LDL enhanced the [^3^H] Thymidine incorporation of macrophages to ~8.88 folds	MSR is an essential and efficient pathway for internalizing lipid PC degradation products in Ox-LDL-induced macrophage proliferation	[35]

Ox-LDL	Murine peritoneal macrophages were treated with 20 μg/mL Ox-LDL for 6 days	Ox-LDL enhanced the [^3^H] Thymidine incorporation of macrophages to ~11.71 folds	Ox-LDL rapidly induces a transient increase in intracellular free calcium ions and activates membrane PKC	[36]

Ox-LDL	Murine peritoneal macrophages were treated with 20 μg/mL Ox-LDL for 7 days	Ox-LDL enhanced the proliferation of macrophages up to ~1.66 folds	Effective endocytosis of lyso-PC of Ox-LDL by macrophages through MSR-AI/AII and subsequent PKC activation led to GM-CSF release into the medium	[37]

Ox-LDL	Murine peritoneal macrophages were treated with 20 μg/mL Ox-LDL for 7 days	Ox-LDL increased the macrophage [^3^H] Thymidine incorporation to 12.1 folds	PI3K is involved in at least part of the downstream signaling pathway, acting after GM-CSF induction	[38]

Ox-LDL	Murine peritoneal resident macrophages and thioglycollate-elicited macrophages were treated with 20 μg/mL Ox-LDL for 7 days	Ox-LDL enhanced the proliferation of resident and elicited macrophages to ~1.79 and ~2.27 folds, respectively	The expression of group-II PLA_2_ in the Ox-LDL-induced macrophages stimulates the GM-CSF release	[39]

Ox-LDL	IFN-gamma/LPS stimulated mouse J774.A1 macrophages were treated with 0–50 μg/mL Ox-LDL for 24 h	20 and 50 μg/mL Ox-LDL promote the proliferation of LPS-interferon-stimulated J774.A1 mouse macrophages to ~1.19 and ~1.2 folds, respectively	By inhibiting the expression of iNOS at both mRNA and protein levels, resulting in decreased production of NO and subsequently NO-induced apoptosis in macrophages	[40]

Urea	1 μg/mL LPS-activated RAW 264.7 macrophages were treated with 0–150 mM urea for 48 h	60, 90, and 150 mM urea promoted the proliferation of LPS-activated RAW 264.7 macrophages to ~1.34, ~1.53, and ~1.63 folds, respectively	By suppressing iNOS-dependent NO production, apoptosis mediated by NO is reduced	[41, 42]

AGEs	RAW 264.7 macrophages were treated with 0–100 μg/mL Glycol-AGEs for 48–120 h	After 120 h culture, 1, 10, and 100 μg/mL Glycol-AGEs promote the proliferation of RAW 264.7 macrophages to ~1.73, ~3.37, and ~2.32 folds, respectively	Via the JAK2-STAT5 pathway	[43]

**Table 4:** Proliferative effects of composition of pathogens and apoptotic cells on macrophages and related mechanisms.

Composition of pathogens and apoptotic cells	Treatment protocols	Results	Mechanisms	References
*Mycoplasma* membranes	Murine BMDMs were treated with heat-inactivated membranes from three *Mycoplasma* species for 3–4 days	The membranes from *M. arginine* and *M. arthritidis* promoted the proliferation of BMDMs on average to ~2.79 and ~3.28 folds, respectively, instead of that from *M. pulmonis* (the specific dosages are not clear)	The production of GM-CSF may be involved	[46]

CpG-B ODNs	RAW 264.7 macrophages were treated with 1 μM CpG-B ODN 1668 for 48 h	CpG-ODN 1668 promoted macrophage proliferation to ~ 1.33 folds	By inducing IL-1Ra and down-regulating the expression of p27	[47]

Apoptotic cells	1.8 × 10^5^ murine BMDMs or human macrophages were incubated with apoptotic Jurkat cells (ACs) at a 1:5 ratio for 45 min. The wells were rinsed with PBS to remove unengulfed ACs, and the macrophages were incubated in a culture medium for 24 h	After 24 h of incubation, ACs increased the cell number of murine BMDMs and human macrophages to ~2.82 and ~2.17 folds, respectively	AC-mediated activation of MerTK-ERK1/2 and activation of DNA-PKcs-mTORC2 by DNase2a-derived AC-oligonucleotides converge to induce Myc and Bhlhe40. Bhlhe40 represses c-Maf to drive macrophage proliferation	[48]

Apoptotic cells	1 × 10^5^ murine BMDMs or human macrophages were incubated with apoptotic Jurkat cells (ACs) at a 1:5 ratio for 45 min. The wells were rinsed with PBS to remove unengulfed ACs, and the macrophages were incubated in a culture medium for 24 h	After 24 h of incubation, ACs increased the cell number of murine BMDMs and human macrophages to ~2.00 and ~1.63 folds, respectively	Apoptotic cells bound to receptors on the surface of macrophages promote glycolysis and the production of lactate, which further bound to GPR132 to activate PKA/AMPK/NAD^+^-NADH/SIRT1/Myc signaling pathway to promote macrophage proliferation	[49, 50]

Apoptotic cells	IC-21 peritoneal macrophage cells were incubated with apoptotic NIH 3T3 cells at a ratio of 1:1 in a trans-well system for 0–48 h	Apoptotic cells induced remarkable M2-like peritoneal macrophage proliferation to ~1.78 folds	Involving PEG_2_, cell cycle, and DNA replication pathways	[51]

**Table 5:** Proliferative effects of natural product extracts on macrophages and related mechanisms.

Natural product extracts	Treatment protocols	Results	Mechanisms	References
Heparin and heparan sulfate	Rat BMDMs were treated with heparin (10 μg/mL) or heparan sulfate (10 μg/mL) for 4 days	Heparin (10 μg/mL) and heparan sulfate (10 μg/mL) induced macrophage proliferation to ~2.04 and ~2.23 folds (DNA content), respectively	Unclear	[52]

Hyalgan	U937 human macrophages were treated with 0.01, 0.1, and 1 mg/mL Hyalgan for 0–168 h	After 168 h, 0.01, and 0.1 mg/mL Hyalgan promoted U937 human macrophage proliferation to ~1.23 and ~1.61 folds, respectively. However, 1 mg/mL Hyalgan inhibited macrophage proliferation by ~99%	Not mentioned	[53]

Ceramide 1-phosphate	Murine BMDMs were treated with 0–100 μM C1P for 24 h	50 μM C1P promoted the proliferation of murine BMDMs to ~1.85 folds	By activating the PI3-K/PKB, ERK, and JNK pathways to stimulate DNA synthesis. GSK-3*β*, c-Myc, cyclin D1, and NF-*κ*B also involved	[54]

Fungal polysaccharides	RAW 264.7 macrophages were treated with polysaccharides (2.5–15 μg/mL) derived from *L. deliciosus* (L. ex Fr.) Gray for 24 h	2.5, 5, 10, and 15 μg/mL polysaccharides increased the proportion of G0/G1 macrophage to ~1.16, ~1.3, ~1.2, and ~1.31 folds compared to the control group, respectively	Janus kinase/signal transducer and activator of transcription, MAPK, chemokine, VEGF, and TGF-*β* may be involved	[55]

Adiponectin	Murine BMDMs were induced to M2 and treated with 0–25 μg/mL recombinant adiponectin for 24 h	25 μg/mL adiponectin promoted cold-induced M2 macrophage proliferation to ~3 folds (assessed by *Ki67* mRNA levels)	Adiponectin binds to T-cadherin on the cell surface of M2 macrophages and promotes cell proliferation by activating PI3K-Akt	[56]

Neuropeptide FF	Murine ATMs were treated with 0.5 nM NPFF for 18 h	After NPFF treatment, the ATM population increased to ~2.24 folds	Probably by increasing *Ndrg2* expression and suppressing the transcription of *Mafb*	[57]

Osteopontin	Murine BMDMs and human monocyte-derived macrophages were treated with 1 μg/mg OPN for 48 h	OPN promotes the proliferation of murine BMDMs and human monocyte-derived macrophages to ~1.64 and ~2.56 folds, respectively	Not mentioned	[58]

**Table 6:** Proliferative effects of gene editing on macrophages and related mechanisms.

Genes	Treatment protocols	Results	Mechanisms	References
*c-myc*	Mononuclear phagocytic cells derived from bone marrow cells were infected by *c-Myc* carrying MRV, and then cultured in the presence of M-CSF (10% L cell-conditioned medium) for 6 days	The number of mononuclear phagocytic cells increased to ~45 folds	*c-Myc* is an oncogene that allows transformed mononuclear phagocytic cells to proliferate extensively in the presence of M-CSF in vitro	[65]

*MafB/c-Maf*	Maf-DKO macrophages were cultured in the presence of M-CSF for different times (0–8 months)	Maf-DKO cells were continuously cultured for more than 8 months without any signs of growth inhibition or morphological changes. For three independent Maf-DKO populations, cell counts over 2 months revealed stable doubling times of 1.44 ± 0.05 days and theoretical amplification factors of 10^11^–10^12^	Continuous proliferation of Maf-DKO macrophages depends on the up-regulation of KLF4 and c-Myc	[66]

## Data Availability

The data that support the findings of this study are openly available in PubMed.
